# 2300 Association between source case cavitation on chest radiograph and QuantiFERON-TB Gold In-Tube conversion among close contacts of active tuberculosis cases in Brazil

**DOI:** 10.1017/cts.2018.45

**Published:** 2018-11-21

**Authors:** Lauren A. Saag, Marcelo Cordeiro-Santos, Afranio Kritski, Bruno Andrade, Solange Cavalcante, Betina Durovni, Megan Turner, Marina Figueiredo, Valeria Rolla, Timothy Sterling

**Affiliations:** 1 Fundação Medicina Tropical Doutor Heitor Vieira Dourado, Manaus, Brazil; 2 Universidade Federal do Rio de Janeiro, Departamento de Clínica Médica, Rio de Janeiro, Brazil; 3 Instituto Brasileiro para Investigação da Tuberculose, Bahia, Brazil; 4 Secretaria Municipal de Saúde do Rio de Janeiro, Rio de Janeiro, Brazil; 5 Division of Infectious Diseases, Department of Medicine, Vanderbilt University, Nashville, TN, USA; 6 Instituto Nacional de Infectologia Evandro Chagas, Rio de Janeiro, Brazil

## Abstract

OBJECTIVES/SPECIFIC AIMS: QuantiFERON-TB Gold In-Tube (QFT) conversion from negative to positive, is regarded as a marker of recent latent tuberculosis infection and may be predictive of incident active tuberculosis (TB) disease. However, it remains unclear how conversion is influenced by individual and environmental factors, including the infectiousness of the source case to whom the contact was exposed. We aimed to examine the effect of infectiousness of TB in the source case, as measured by presence of cavitation on chest X-ray, on the incidence of QFT conversion among close contacts of the pulmonary TB index case, after adjusting for potential confounding by contact and source case characteristics. METHODS/STUDY POPULATION: The Regional Prospective Observational Research for Tuberculosis (RePORT)-Brazil is an ongoing prospective cohort study that enrolls close contacts of culture-confirmed pulmonary TB patients and follows them for 24 months for development of active TB. Demographic, clinical, and diagnostic information are obtained at baseline and during follow-up at clinical visits and by telephone. QFT testing is performed at baseline and repeated after 6 months if the baseline QFT is negative. A positive IFN-γ value is defined as >0.35 IU/mL, as recommended by the manufacturer and the CDC, and QFT conversion is defined as a negative QFT at baseline followed by a positive QFT at 6 months. RESULTS/ANTICIPATED RESULTS: Among 260 enrolled contacts with nonpositive baseline QFT results and 6 months of follow-up, 198 (76%) were retested with QFT 6 months after enrollment. Of those retested, 26 (13%) converted to positive. Presence of any cavitation in the source case, based on chest radiography, was significantly associated with QFT-conversion (ORunadjusted=2.4, 95% CI: 1.0–5.7). Additional univariate analyses revealed that QFT conversion was associated with black and brown race (compared with white race) of the contact, current smoking and current alcohol use in the source case. After adjusting for potential confounders (age, sex, and race of the contact and current smoking of the source case), the association between source case cavitation and QFT conversion remained (ORadjusted=2.5 95% CI: 1.0–6.2). As of December 6, 2017, none of the QFT-retested contacts had developed active TB, with a median follow-up of 12.3 months (IQR: 7.1–13.1). We anticipate that ongoing enrollment and follow-up may yield cases of active TB; future analyses will provide greater precision for examining predictors of QFT-conversion and its association with incident TB. DISCUSSION/SIGNIFICANCE OF IMPACT: Our preliminary results agree with published literature suggesting the infectiousness of TB in the index case is a predictor of incident LTBI. Along with recent LTBI, immune suppression, HIV co-infection, and type 2 diabetes are considered risk factors for progression to active TB disease. Because only a small proportion of persons progress from LTBI to active TB disease, it is not appropriate to treat all persons with LTBI. Thus, more research is needed to identify groups at highest risk for QFT-conversion and incident TB disease, so these groups can be targeted for TB prevention, interventions, and facilitate a decline in TB incidence and mortality.

